# Hospital Meetings

**Published:** 1905-03-04

**Authors:** 


### HOSPITAL MEETINGS.

### KING'S COLLEGE HOSPITAL.

The Rev. Dr. Headlam, Principal of King's College, in presiding over the 66th annual Court of the Corporation on Thursday, 22nd ult., made an important statement with regard to the removal of the hospital to Camberwell. He stated that, notwithstanding opposition from the Westminster City Council, the necessary Act of Parliament had been obtained, almost all the negotiations with regard to the site had been completed, and six selected architects were at work engaged in a limited competition, with Mr. Roland Plumbe as architectural assessor; their plans would be sent in at the end of March, when the selection would be made and the work entered upon without delay. He hoped it would be possible to lay the foundation-stone during the present year. In one respect, the site was even better than had been anticipated, since a movement had been undertaken by the residents of Denmark Hill and the local Borough Councils by which a plot of 25 acres adjoining would, it was hoped, be secured for all time as a public park, thus greatly improving the outlook. With regard to funds, in a year generally admitted to be unfavourable to charitable purposes, they had no cause for complaint. Including the value of the site, they had £133,000 in hand, as the result of something over a year's work. Even during the last few months, a considerable number of donations had been coming in. The Drapers' Company had doubled their donation, making it £10,000, on condition that the entire sum was raised during 1905. The grant of £2,500 towards the building fund had been made by King Edward's Hospital Fund, and, although the neighbourhood to which they were removing was by no means a wealthy one, the matter was being taken up with sufficient interest to greatly encourage the committee.

Referring to the report issued by the committee appointed by King Edward's Hospital Fund to inquire into the expenditure on the medical schools of hospital funds, the chairman said the Governors would be glad to have public endorsement of the policy they had always pursued (not for lack of funds but believing it to be the right one) in the statement unhesitatingly made by that committee, that during 1903, King's College Hospital did not, directly or indirectly, subsidise its medical school.

The report and accounts having been adopted, and other business transacted, the meeting closed with a vote of thanks to the chairman.

### TOTTENHAM HOSPITAL, N.

The annual general meeting of subscribers and donors was held in the hospital last Tuesday. Sir Francis Cory-Wright was in the chair. After the reading of the circular convening the meeting, and confirming the minutes of the last meeting, the annual report was read. The Chairman, in his speech, alluded to the continued generosity of King Edward's Fund, and spoke of the indebtedness of the hospital governors to the Ladies' Association for the material assistance rendered. He was happy to say that the deficit was lower than last year. He went on to refer to the death of Dr. Gilbart-Smith, and stated that his vacancy would be filled by Sir J. Reed. A hearty vote of thanks was moved to the honorary staff of physicians and surgeons, and the usual votes of thanks ended the meeting.

### SEAMEN'S HOSPITAL SOCIETY.

The eighty-fourth annual meeting of the Seamen's Hospital Society, under the chairmanship of Mr. Percival Nairne, was held at the new offices, 13a Cockspur Street, W., on Friday, 23rd ult.

In welcoming the supporters of the Society to the new offices, the chairman said it was hoped that by taking premises in the West End, the work might become better known and, consequently, better supported. They were glad to find themselves near their old friends, the Shipwrecked Mariners' Society, who rendered much assistance by sending patients to their homes, and whose representative he was very glad to see present. The very best advertisement the Seamen's Society ever had was the old ship in the Thames, and it was intended now to do something in the way of advertisement by placing models in a conspicuous position in the windows, with a view to bringing the Society before residents and others in the West End. With reference to the year's work, he could not say there had been freedom from financial anxiety, since again the income from charitable sources had been insufficient to meet the ordinary expenditure, and it had been necessary to encroach upon the reserve fund. He feared that the idea still held ground that, the hospital being housed in a Government building, it was in part supported by the State, and that this impression perhaps diverted legacies and donations from the Society. While, however, a considerable additional annual income was needed to meet current expenses, it was gratifying to note that, with the exception of ships' collections, nearly every item of receipt showed an increase. From legacies a larger amount had been received than had been the case for some years, and a further munificent contribution had been received from the Elder Brethren of Trinity House, London. In recognition of the services rendered by that corporation ever since 1821, the committee had erected in their branch hospital a tablet naming one of the beds the "Trinity House Bed." King Edward's Hospital Fund, in addition to a grant of £500, had given a special donation for this year of £1,000. The total ordinary income was £15,783 16s. 5d., while the ordinary expenditure was £20,578 8s. 7d., giving an adverse balance of £6,648 1s. 6d. The new out-patient department at the Branch Hospital had been completed at a cost of £3,020, and had been in occupation since June, proving a valuable and useful acquisition to the society's work. Negotiations with regard to the taking over of the Passmore Edwards Cottage Hospital at Tilbury had been carried on during the year, the matter, however, was still in abeyance.

### The London School of Tropical Medicine.

He was glad to be able to say that the London School of Tropical Medicine continued to progress. Being little advertised it was little known, the Liverpool School being much more before the public eye. It should, however, be remembered that the idea of a Tropical School of Medicine was initiated in London, and that, while the Liverpool School was doing most excellent work in research, the London School, without neglecting that department, was *par excellence* a teaching body for students intending to proceed to the Tropics. There could be no question as to the importance to the Empire of the kind of instruction afforded by the London School, which during the last twelve months passed 115 students through the curriculum, most of them remaining for the entire course of three months. The Governments of the Crown Colonies and of India, recognising the debt they owed to the School, had contributed from a fund at their disposal £1,000 per annum for five years, for the teaching of Protozoology and Helminthology, and a further sum of £250 for fitting up a laboratory had also been given. It was believed that the second mission of Sir Francis Lovell, who returned to England in June, had been the means of widely advertising the school and bringing it many new friends. The Craggs Research Prize for 1904 had been awarded to Mr. J. Catto, M.B., Ch.B., for his discovery in the human body of a worm, "Schistosoma S. Cattoi." Having referred to the banquet at the Hôtel Cecil on May 10, at which Mr. Joseph Chamberlain, to whom the school owes its inception, has undertaken to preside, the chairman moved the adoption of the report and accounts, and, Mr. Swanzy having seconded the motion, it was carried unanimously.

Other business having been transacted, a vote of thanks to Mr. Nairne closed the meeting.

### CANCER HOSPITAL, FULHAM ROAD, S.W.

The annual meeting of the Governors of the Cancer Hospital was held in the Board-room of the hospital last week. Lord Ludlow presided, and moved the adoption of the annual report, which was seconded by Colonel T. R. Parr, and carried. Mr. Hooper, in moving the thanks of the meeting to the honorary staff, the chaplain, and ministers of other denominations, referred especially to the Rev. Farringdon Downes, who, he said, had undertaken to connect by telephone the chapel and also the entertainment-room with each bed in the hospital.

Mr. Jessett, F.R.C.S., supporting the motion, said that it was the anniversary of the establishment of the hospital in Fulham Road. Twenty-one years ago it was moved from Piccadilly. In 1884 the staff was increased, and, since then, the work had gone on steadily. The hospital had now a perfectly equipped pathological department. A new feature was the appointment of a surgical registrar, which the committee hoped would be a very useful step. Another point was the suggestion that a surgeon and pathologist should be sent to Paris to study Dr. Doyen's methods and bring away some of his serum to be used at Fulham.

Mr. Jessett then moved a vote of thanks to donors of useful gifts for the patients; to the lady visitors; to all friends and supporters of the hospital; to town and country bankers who have kindly received contributions; also to the metropolitan and provincial Press. This was seconded by General Bailie, and carried. A hearty vote of thanks to the chairman closed the meeting.

### ROYAL LONDON OPHTHALMIC HOSPITAL.

The annual meeting of the Royal London Ophthalmic Hospital was held in the Board-room on Thursday in last week, Lord Avebury, the president of the Hospital, being in the chair.

The minutes and report of the year's work were read by the secretary, Mr. R. J. Bland, and confirmed by the chairman, who then moved the adoption of the report, which was carried. He said that it was the centenary of the hospital, and that the number of patients was greater than had ever been before. He was, however, sorry to say that 20 beds were closed for want of funds. The income, it was true, had increased, but the difference was very small. The donations last year amounted to £300 more than the year before. Still they were lower than they had been in the past. The committee, however, gratefully acknowledged donations from the Hospital Sunday Fund, Hospital Saturday Fund, and King Edward's Hospital Fund. The annual course of the hospital work had suffered from increase of rates to £996 6s. 11d. which was much heavier than most hospitals in London. The committee with deep regret record the death of the Duke of Cambridge, who had been a patron of the hospital since 1858. They had to regret the losses they had sustained by the retirement of Mr. Warren Tay, 27 years a surgeon of the hospital, and by the death of Mr. Arthur Quarry Silcock, who spent 18 years upon the staff. The committee was very grateful for the liberal donation of £750 from the Goldsmiths' Company, of which £500 was for the Centenary Fund and £250 for the general account.

Mr. Sturgis, in seconding the motion, spoke of the economy exercised in the hospital, and, as an instance, said that during the last six months a special committee had been investigating the management of the drug and dispensary stores, and that it was satisfactory to find that, though the number of patients had increased, the expenditure had decreased. Another point they had to deal with was the question as to the abuse of hospitals. It was very difficult to discriminate between those who could afford to pay and those who could not. In this hospital, however, they had a double check, since each patient on arrival in the out-patient department was interviewed by the assistant secretary, who, if he had suspicions, passed the patient on to the inquiry officer. A further point to be mentioned was the Centenary Fund, hitherto called the Rent Fund. For this they had made an appeal for £50,000, and had only received £5,000.

The question of the high rating of the hospital arose later during the meeting, and it was stated that the committee intended to make an appeal for a reduction.

A hearty vote of thanks to the chairman ended the proceedings.

### NATIONAL ORTHOPÆDIC HOSPITAL.

The last annual meeting of the National Orthopædic Hospital as a separate institution took place last week, the Duke of Marlborough, president, in the chair.

The Chairman having formally moved the adoption of the report and accounts for 1904, Mr. J. R. Cooper said the report showed that another year of increased work had been accomplished. The vitality of the institution was shown by the increased amount received in subscriptions, the figures being £783 15s., as compared with £437 5s. in the previous year. Donations, however, were considerably less than in 1903. Patients' payments, on the other hand had increased, being £1,170 4s. 6d. as compared with £794 8s. 5d. The scheme of amalgamation with the Royal Orthopædic Hospital was now practically completed, the signature of his Majesty the King having, he believed, just been put to the necessary charter. The increase in the staff, which now consisted of six surgeons instead of four, had necessitated a heavy expenditure in providing a new operating theatre, which also reduced the space, leaving room for only 52 beds and cots instead of 60. The total expenditure, however, was less than in 1903, being £3,442 11s. 3d., as compared with £3 511 4s. 4d. He hoped that those who agreed with the council of King Edward's Fund, that unity was strength, would devote themselves with as much earnestness and zeal to the work, when fusion was completed, as they had done in the past, and in this connection he wished especially to emphasise the devotion of the surgical, medical, and nursing staff.

The report and accounts having been adopted, Mr. E. Muirhead Little and Mr. T. Openshaw spoke of the benefits received by the children from their stay of several weeks at Blenheim Park, owing to the kindness of the Duke and Duchess of Marlborough, who in this and many other ways showed their interest in the hospital.

The Chairman, replying to a vote of thanks, said he had been much struck that afternoon, in going through the wards, by the exceedingly beneficial work of instruction being carried on by two lady teachers under the London County Council, the hospital having been adopted as an invalid school centre. He greatly regretted that, this being the last meeting of the National Orthopædic Hospital as a separate body, it was necessary for him to resign the office of president. He hoped, however, that the work of the amalgamated hospitals would go forward, and be even greater than that which had been done in the past.

### THE AFTER CARE ASSOCIATION.

The Bishop of St. Albans, in presiding at the annual meeting of the After Care Association last week, strongly urged its claims on the support of the public, if on no other grounds, as an agency which had a preventive influence. The prevention of relapse after release from an institution was in itself a great work, and he thought it was worthy of much greater financial aid than it had yet received, since it also prevented a large amount of intemperance, and enabled a number of men and women every year to take their place among sane and civilised people. During the year there had been 255 cases before the council —168 women and 87 men. While the income of £818 12s. 2d was an increase of £170 8s. 7d. on that of the previous year, it was quite insufficient to carry on the invaluable work of the Association. The boarding out of patients had been continued with success, and a new departure had been made in the formation of local branches. The Association had to deplore the death, during the year, of the founder, the Rev. Henry Hawkins, whose writings had done so much to make the work known, and whose presence at the meetings had always been of great help and encouragement. The report and accounts having been passed, Dr. F. Needham, Dr. G. H. Savage, the Rev. H. Stephens, and others addressed the meeting.

